# Anthropometric and cardiometabolic risk factors in parents and child obesity in Segamat, Malaysia

**DOI:** 10.1093/ije/dyx114

**Published:** 2017-06-29

**Authors:** Uttara Partap, Elizabeth H Young, Pascale Allotey, Manjinder S Sandhu, Daniel D Reidpath

**Affiliations:** 1 Department of Medicine, University of Cambridge, UK; 2 Wellcome Trust Sanger Institute, Hinxton, UK; 3 Jeffrey Cheah School of Medicine and Health Sciences, Monash University Malaysia, Selangor, Malaysia; 4 South East Asia Community Observatory, Segamat, Malaysia

**Keywords:** South East Asia, child obesity, anthropometric risk factors, cardiometabolic risk factors, intergenerational associations, health and demographic surveillance

## Abstract

**Background:**

There is little evidence regarding risk factors for child obesity in Asian populations, including the role of parental anthropometric and cardiometabolic risk factors. We examined the relation between parental risk factors and child obesity in a Malaysian population.

**Methods:**

We used data from health and demographic surveillance conducted by the South East Asia Community Observatory in Segamat, Malaysia. Analyses included 9207 individuals (4806 children, 2570 mothers and 1831 fathers). Child obesity was defined based on the World Health Organization 2007 reference. We assessed the relation between parental anthropometric (overweight, obesity and central obesity) and cardiometabolic (systolic hypertension, diastolic hypertension and hyperglycaemia) risk factors and child obesity, using mixed effects Poisson regression models with robust standard errors.

**Results:**

We found a high burden of overweight and obesity among children in this population (30% overweight or obese). Children of one or more obese parents had a 2-fold greater risk of being obese compared with children of non-obese parents. Sequential adjustment for parental and child characteristics did not materially affect estimates (fully adjusted relative risk for obesity in both parents: 2.39, 95% confidence interval: 1.82, 3.10, *P* < 0.001; *P* for trend < 0.001). These associations were not modified by parental or child sex. We found no consistent evidence for associations between parental cardiometabolic risk factors and child obesity.

**Conclusions:**

Parental obesity was strongly associated with child obesity in this population. Further exploration of the behavioural and environmental drivers of these associations may help inform strategies addressing child obesity in Asia.

## Introduction

The increasing global prevalence of child obesity is a public health issue of growing concern.^1^ Obesity among children is associated with multiple adverse consequences, ranging from psychological morbidity during childhood to earlier development of cardiometabolic diseases in adulthood.^1–6^ A comprehensive understanding of the underlying risk factors is essential to inform well-designed preventive and management efforts. Thus far, a notable part of child obesity research has been focused on investigating intergenerational associations of body mass index (BMI), generally in European or North American populations.^7–29^ Findings reported in such studies have been inconsistent, particularly regarding the modifying effect of parental or child sex.^7–29^ Few studies have examined the relation between other cardiometabolic risk factors in parents and child obesity. This is an important issue to clarify given the interlinked nature of both anthropometric and cardiometabolic risk measures.^30–32^

Importantly, little evidence regarding intergenerational influences on child obesity has been generated from Asia. In 2013, the number of obese children in the region exceeded that in all higher-income countries combined, and child obesity prevalence in certain Asian countries was comparable to estimates from European nations.^33^^,^^34^ This is widely understood to be the result of changing lifestyles that have accompanied economic transitions in Asian countries.^35^^,^^36^ Region-specific research is required to inform suitable strategies to address the growing burden of child obesity in Asia, where intergenerational influences on child BMI might be distinct from those observed in other populations.^37^^,^^38^ We sought to address the current gap in evidence from Asia by examining the relation between parental anthropometric and cardiometabolic risk factors and child obesity in a population living in Segamat, Malaysia.

## Methods

### Study population

We used data from the South East Asia Community Observatory (SEACO), a health and demographic surveillance system (HDSS) in southern peninsular Malaysia. SEACO operates within five sub-districts of Segamat district in the state of Johor.^39^ It conducts annual enumeration of all consenting households and individuals within its defined area, during which it collects basic household and individual level socio-demographic data. It has also undertaken a health survey on individuals aged 5 years and above, using methods adapted from standardized health data collection tools.^40–44^ All information is collected on encrypted tablets, and participant data are linked across surveys. Ethical approval for surveys is obtained from the Monash University Human Research Ethics Committee.

For this analysis, we obtained data from the baseline enumeration (completed 2013) and the health survey (completed 2014). Using enumeration data, parent-child trios or pairs were identified and information on the child’s birth order was obtained. All other information was obtained from the health survey. This included biophysical measurements taken by trained data collectors: height and weight data for children aged 6–19 years and for their parents (measured using a Transtek digital weighing scale with height gauge, model GBS-721), and parents’ waist circumference (AccuFitness Myotape), blood pressure (Omron automated blood pressure monitor, HEM-7203) and random blood glucose (Omron blood glucose monitoring system, HGM-111). Information on: the child’s age and ethnicity; and parents’ age, highest level of education attained or currently attaining, and employment in the past 30 days was also obtained. Pairs or trios where the mother or daughter was pregnant were excluded. The resulting cross-sectional dataset used for analysis comprised parents’ and children’s current biophysical and socio-demographic measures as collected at the time of the health survey.

### Definitions

In accordance with World Health Organization (WHO) guidelines, children were defined as individuals aged 19 years or below.^45^ BMI was calculated as weight divided by the square of the height, and was expressed as age-adjusted z-scores using the sex-specific WHO 2007 BMI reference for children aged 5–19. This reference classifies overweight and obesity as BMI for age greater than one and two standard deviations above the mean, respectively.^45^^,^^46^ For parents, overweight was defined as BMI from 25 kg/m^2^ to less than 30 kg/m^2^, and obesity as 30 kg/m^2^ or more.^47^ Central obesity was defined according to ethnicity-specific International Diabetes Federation (IDF) recommendations, as waist circumference greater than or equal to 90 cm in men and 80 cm in women.^48^ For systolic and diastolic blood pressure, the mean of the final two of three readings was used. Systolic and diastolic hypertension were defined as mean systolic and diastolic blood pressure greater than or equal to 140 mmHg and 90 mmHg, respectively.^49^ Hyperglycaemia was defined as random blood glucose greater than or equal to 11.1 mmol/l.^50^

### Statistical analysis

We examined children’s and parental characteristics by category of child obesity (non-obese, including overweight, versus obese). Continuous variables were compared using Student’s t test, and categorical variables were compared using Pearson’s chi-square test or Fisher’s exact test. For variables with more than 5% of observations missing, we compared child and parental characteristics for individuals with missing versus non-missing data. Following this, univariable linear regression models were used to examine the relationship between each parental risk measure, examined as a continuous variable, and child BMI z-score. Likelihood ratio tests with child BMI as a continuous and binary outcome were used to check departure from linearity. Univariable associations between potential confounders (child’s ethnicity and birth order, and parental age, education and employment) and parental exposures and child BMI z-score were also examined in linear regression models, and departure from linearity was checked where appropriate, as above.

We then used mixed effects Poisson regression with robust standard errors to estimate the risk for child obesity associated with one (either the mother or the father) or both parents having a specific anthropometric (overweight, obesity and central obesity) or cardiometabolic (systolic and diastolic hypertension, and hyperglycaemia) risk factor. Poisson regression was considered the most suitable method of analysis: (i) given the cross-sectional nature of our data, which included no staggered or repeated measures; and (ii) in order to provide more accurate measures of risk, as opposed to logistic regression, given the high prevalence of the outcome of interest (child obesity) observed in the population.^51–53^ Mixed effects models with robust standard errors were used in order to account for the clustered nature of the data, which included multiple parent-child pairs or trios from the same household.^52^ Each regression model was based on analysis of complete records. Each risk factor was examined in a separate series of models, beginning with univariable models assessing crude associations, and then making sequential adjustments for potential confounders. Fully-adjusted models included the following variables: child’s birth order and ethnicity, and both parents’ age, education and employment. Models exploring systolic and diastolic hypertension were further adjusted for both parents’ BMI and random blood glucose. Models exploring hyperglycaemia were additionally adjusted for both parents’ BMI and systolic blood pressure. Continuous variables included as covariates were not categorized. We explored the addition of random effects to models to account for clustering at the household and sub-district levels. Sub-district level clustering was found to be minimal; all final models were therefore adjusted for clustering at the household level only.

Following this, we explored potential effect modification by parental or child sex. First, we stratified the models described above by child sex, in order to explore potential differences in the magnitude of associations between sons and daughters. We then constructed a separate set of regression models with child obesity as the outcome of interest, and anthropometric and cardiometabolic risk factors in each specific parent as the primary exposures, to compare the magnitude of associations between mothers and fathers. Models were adjusted as described above for covariates and for clustering. We further stratified these models by child sex to assess potential differences in effect size. Finally, we assessed interaction between the parent’s or child’s sex and the parental risk factor of interest in these models, using likelihood ratio tests.

To explore the consistency of results obtained, we repeated analyses using mixed effects logistic regression models. We also constructed mixed effects linear regression models with robust standard errors examining each parent’s risk measures as continuous exposures (BMI, waist circumference, systolic and diastolic blood pressure and random blood glucose), and child BMI z-score as the outcome. Models were constructed in the same manner as described above, with the final models including the same covariates and adjusted for clustering at the household level. Analyses were performed using Stata 13 and Stata 14 (StataCorp, TX).

## Results

A total of 9207 individuals who had information on current biophysical and socio-demographic measures of interest were included in this study (4806 children, 2570 unique mothers and 1831 unique fathers). Children covered by the health survey but not included in these analyses were similar to those who were included, and adults were comparable in terms of risk factors (data not shown). Analyses covered 3510 trios and 1296 additional mother-child pairs. Children included in mother-child pairs were similar to those included as part of trios (data not shown). Missing data reached approximately 10% for maternal systolic and diastolic blood pressure and random blood glucose. There was no consistent trend with respect to other anthropometric and cardiometabolic measures, and no difference in child BMI z-score, among mothers with missing versus non-missing data on these measures (Supplementary Table S1, available as Supplementary data at *IJE* online).

Overall, 16.3% of children were overweight, not including obese, and 14.3% were obese. The prevalence of indices of obesity among parents was similarly high: 27.9% of mothers and 16.6% of fathers were obese, and 69.8% of mothers and 41.2% of fathers were centrally obese. Approximately 10% of parents were hyperglycaemic, and up to 24% had systolic or diastolic hypertension (Tables 1 and 2). Risk factors were clustered within households and correlated between mothers and fathers (*P* for all Pearson’s correlation coefficients < 0.001) (Supplementary Tables S2 and S3, available as Supplementary data at *IJE* online). Children of parents who were overweight, obese, hyperglycaemic or with systolic hypertension were more likely to be obese (Table 2; Supplementary Table S4, available as Supplementary data at *IJE* online).

**Table 1.** Study population characteristics

	Children		Mothers		Fathers	
*N*	4806		2570		1831	
Sex, *n* (%)						
Male	2362	(49.2)				
Female	2444	(50.8)				
Age, years, mean (SD)	12.7	(3.8)	47.2	(10.3)	50.6	(10.2)
Ethnicity, *n* (%)						
Malay	3256	(67.8)	1698	(66.1)	1240	(67.7)
Indian	466	(9.7)	261	(10.2)	208	(11.4)
Chinese	948	(19.7)	495	(19.3)	331	(18.1)
Bumiputera/Orang Asli	86	(1.8)	56	(2.2)	32	(1.8)
Other	32	(0.7)	15	(0.6)	5	(0.3)
Missing	18	(0.4)	45	(1.8)	16	(0.9)
Birth order, *n* (%)						
1	2049	(42.6)				
2	1467	(30.5)				
3	792	(16.5)				
4	324	(6.7)				
5	121	(2.5)				
6	36	(0.8)				
7+	17	(0.4)				
Body mass index						
Mean (SD), kg/m^2^ or z-score^a^	0.39	(1.5)	27.4	(5.3)	26.1	(4.5)
Overweight, *n* (%)	785	(16.3)	873	(34.0)	724	(39.5)
Obese, *n* (%)	689	(14.3)	716	(27.9)	303	(16.6)
Waist circumference						
Mean (SD), cm			86.6	(10.9)	89.0	(10.4)
Centrally obese, *n* (%)			1793	(69.8)	755	(41.2)
Systolic blood pressure						
Mean (SD), mmHg			129.1	(18.4)	131.7	(17.0)
Systolic hypertension, *n* (%)			521	(20.3)	432	(23.6)
Diastolic blood pressure						
Mean (SD), mmHg			78.8	(10.7)	79.3	(10.8)
Diastolic hypertension, *n* (%)			356	(13.9)	279	(15.2)
Random blood glucose						
Mean (SD), mmol/l			7.9	(3.6)	7.9	(3.3)
Hyperglycaemic, *n* (%)			232	(9.0)	162	(8.9)
Education level attained, *n* (%)						
None			67	(2.6)	20	(1.1)
Attended/completed primary			758	(29.5)	563	(30.8)
Attended/completed secondary			1542	(60.0)	1122	(61.3)
Attended/completed tertiary/diploma			91	(3.5)	82	(4.5)
Missing			112	(4.4)	44	(2.4)
Employment in past 30 days, *n* (%)						
Student/not working			161	(6.3)	117	(6.4)
Casual/part-time			128	(5.0)	88	(4.8)
Full time			455	(17.7)	884	(48.3)
Self-employed/housewife			1805	(70.1)	610	(33.3)
Retired			22	(0.9)	127	(6.9)
Missing			3	(0.1)	5	(0.3)

^a^Mean body mass index for children is reported as age- and sex-adjusted z-scores, using the World Health Organization 2007 reference.

**Table 2.** Proportion of children obese, by child or parental characteristic (*N* = 4806)

	Child			Mother			Father		
Child or parental characteristic	Obese	(*n*, %)	*P*	Obese	(*n*, %)	*P*	Obese	(*n*, %)	*P*
*N*	689	(14.3)							
Sex									
Male	400	(16.9)							
Female	289	(17.8)	<0.001						
Age category, years									
5–9	222	(18.9)							
10–14	288	(15.7)							
15–19	179	(10.0)	<0.001						
Ethnicity									
Malay	485	(14.9)							
Indian	61	(13.9)							
Chinese	119	(12.6)							
Bumiputera/Orang Asli	16	(18.6)							
Other	6	(18.8)	0.227						
Missing	2	(11.1)							
Birth order									
1	299	(14.6)							
2	200	(13.6)							
3	120	(15.2)							
4+	70	(14.1)	0.763						
Overweight or obese									
No				165	(9.3)		158	(10.8)	
Yes				226	(13.8)	<0.001	195	(14.2)	0.001
									
Obese									
No				344	(11.8)		353	(12.4)	
Yes				236	(20.7)	<0.001	114	(19.8)	<0.001
Centrally obese									
No				95	(8.8)		228	(12.2)	
Yes				467	(16.1)	<0.001	227	(15.6)	0.005
Systolic hypertension									
No				399	(13.6)		328	(12.6)	
Yes				126	(16.6)	0.035	129	(17.0)	0.002
Diastolic hypertension									
No				435	(13.9)		371	(13.1)	
Yes				91	(15.6)	0.294	84	(16.1)	0.061
Hyperglycaemic									
No				457	(13.4)		408	(13.2)	
Yes				73	(22.1)	<0.001	52	(17.9)	0.028
Education level attained									
None				19	(14.4)		4	(13.3)	
Attending/completed primary				170	(13.6)		117	(12.3)	
Attending/completed secondary				437	(14.4)		307	(13.6)	
Attending/completed tertiary/diploma				35	(18.8)	0.317	36	(21.2)	0.021
Missing				28	(13.9)		111	(14.6)	
Employment in past 30 days									
Student/not working				47	(16.2)		34	(15.1)	
Casual/part time				44	(17.9)		29	(17.1)	
Full time				146	(16.4)		226	(13.0)	
Self-employed/housewife				445	(13.3)		153	(13.4)	
Retired				6	(17.1)	0.055	35	(16.1)	0.406
Missing				1	(25.0)		98	(14.4)	

*P*-values are reported for differences in proportions across variable categories, between non-obese and obese. Pearson’s chi-square and Fisher’s exact tests were used to compare categorical variables (Fisher’s exact for categories with cell frequencies of less than five).

We explored the relation between parental anthropometric and cardiometabolic risk factors and child obesity using Poisson regression. In unadjusted models, children having one (either the mother or the father) or two parents with obesity or central obesity had a 2-fold greater risk of obesity compared with children of non-obese parents (Supplementary Table S5, available as Supplementary data at *IJE* online). Sequential adjustments for child and parental characteristics, such as age, ethnicity or employment, did not notably affect estimates [fully adjusted risk ratio (RR) for obesity in both parents: 2.38, 95% confidence interval (95% CI): 1.82, 3.10, *P* < 0.001; for central obesity in both parents: 2.18, 95% CI: 1.55, 3.07, *P* < 0.001] (Table 3, Supplementary Table S5). The linear increase in risk of child obesity was approximately 1.5 for each additional parent (either mother or father) being obese or centrally obese (*P* < 0.001 for both risk factors) (Supplementary Table S7, available as Supplementary data at *IJE* online). We did not observe strong or consistent associations between any other parental risk factor and child obesity (Table 3; Supplementary Table S5). Furthermore, whilst crude analyses indicated that children of fathers who were attending or had completed tertiary education were more likely to be obese (*P* = 0.021; Table 2), we found no evidence of association between child obesity and either parent’s education or employment in regressions (data not shown).

**Table 3.** Risk ratios for child obesity associated with number of parents having a specific anthropometric or cardiometabolic risk factor

		One parent^a^		Both parents	
Parental risk factor	*N*	Risk ratio (95% confidence interval)	*P*	Risk ratio (95% confidence interval)	*P*
Overweight	2003	1.12 (0.80, 1.58)	0.513	1.54 (1.06, 2.24)	0.025
Obese	3149	1.44 (1.15, 1.80)	0.001	2.38 (1.82, 3.10)	<0.001
Centrally obese	3032	1.62 (1.14, 2.29)	0.007	2.18 (1.55, 3.07)	<0.001
Systolic hypertension^b^	2762	1.22 (0.96, 1.55)	0.101	1.45 (1.02, 2.07)	0.039
Diastolic hypertension^b^	2782	1.10 (0.87, 1.39)	0.434	1.20 (0.83, 1.72)	0.339
Hyperglycaemic^c^	2762	1.36 (1.02, 1.81)	0.035	2.28 (1.27, 4.12)	0.006

Models were adjusted for child’s ethnicity, birth order and maternal and paternal age, education and employment.

^a^One parent refers to either the mother or the father having the specific risk factor.

^b^Models exploring systolic and diastolic blood pressure also included adjustments for maternal and paternal BMI and random blood glucose.

^c^Models exploring random blood glucose also included adjustments for maternal and paternal BMI and systolic blood pressure.

**Figure 1.**
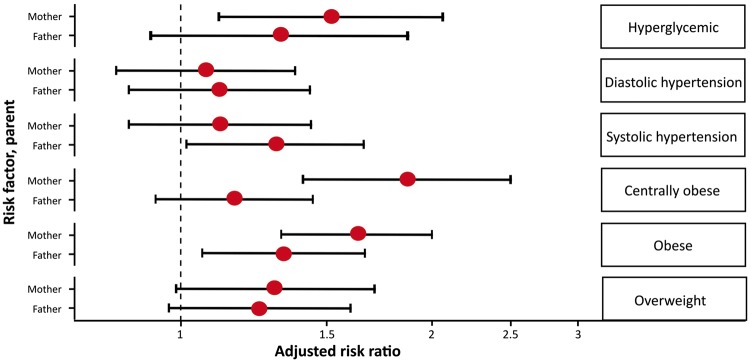
Risk ratios for child obesity associated with maternal and paternal anthropometric and cardiometabolic risk factors. Error bars represent 95% confidence intervals.

Previous evidence has suggested a modifying effect of parental and child sex on associations between parental and child anthropometric measures.^9^^,^^13–16^^,^^21^^,^^25^ We assessed evidence for such interdependency in our data. In analyses stratified by child sex, there was no consistent difference in magnitudes of association between parental anthropometric or cardiometabolic risk factors and child obesity (Supplementary Tables S6 and S9, available as Supplementary data at *IJE* online). Similarly, we observed no differences in associations when stratifying analyses by parental sex (Figure 1; Supplementary Tables S8 and S9, available as Supplementary data at *IJE* online). There was no difference between fully adjusted models with and without additional terms for interaction of parental or child sex with parental risk factors of interest (Supplementary Table S10, available as Supplementary data at *IJE* online).

For all analyses, estimates from logistic and linear regression models were consistent with results from Poisson regression models (Supplementary Tables S11 and S12, available as Supplementary data at *IJE* online; data not shown for logistic regression models).

## Discussion

In this study, we present evidence of independent associations between parental anthropometric risk factors and child obesity in a Malaysian population. Compared with children of non-obese parents, children of two parents who were obese or centrally obese had an approximately 2-fold increased risk of obesity. We found no consistent evidence of associations between parental cardiometabolic risk factors and child obesity, and no statistical evidence of effect modification by parental or child sex. Our findings emphasize the importance of better understanding the underlying pathways for these intergenerational associations; they have potential implications for the design of strategies to address child obesity. This is particularly relevant in the context of this population, given the high burden of child overweight and obesity.

Our results support and extend previous research on intergenerational associations in anthropometric indices,^7–29^ providing evidence of increased child obesity risk with each additional parent being obese, and suggesting no interactive effect of parental or child sex on these associations. Some previous studies have reported similar patterns in other cardiometabolic risk measures.^14^^,^^16^^,^^22–25^^,^^29^^,^^54^ Only one reviewed here directly examined the relation between parental diabetes status and child obesity, and two reported a notable contribution of components of metabolic syndrome among parents to similar measures among their children, including BMI.^55–57^ Unlike these studies, we found no clear evidence of contribution of parental cardiometabolic risk factors to child obesity risk. Furthermore, the evidence regarding effect modification by parental or child sex is equivocal. Whereas some studies have suggested a marginally greater maternal influence,^9^^,^^14^^,^^15^^,^^17^^,^^19^^,^^26^^,^^28^ a 2-fold greater risk for daughters compared with sons,^25^ or strongly assortative (mother-daughter and father-son) associations,^21^ many other studies have found no evidence of interaction.^8^^,^^10–12^^,^^16^^,^^18^^,^^20^^,^^24^^,^^29^ Few studies have explored the cumulative influence of both parents’ risk factor status on child obesity risk.^13^^,^^19^^,^^28^ Importantly, most of the existing evidence is derived from North American or Western European populations,^7–11^^,^^13–24^^,^^26–28^ with little evidence from Asia.^12^^,^^25^^,^^29^ Our findings provide valuable insights into these relationships within a large Asian population, forming a basis for further research within this region.

Our evidence also suggests that aspects of the family environment, such as shared family behaviours relating to nutrition or physical activity, may explain to a large extent the relation between parental anthropometric risk factors and child obesity. We observed similar magnitudes of association between maternal versus paternal risk factors and child obesity in this study. Risk measures were also notably correlated between mothers and fathers, and clustered within households from which the study population was derived. Many studies have reported associations between parental and child physical activity and nutritional habits,^58^^,^^61^ with some evidence from Asian populations suggesting that such behaviours strongly relate to family obesity status.^12^ These and other such shared behaviours may similarly underlie the associations observed in this population.

Other mechanisms underlying intergenerational associations in obesity have also been proposed. These include intrauterine influences, such as exposure to excess maternal adiposity or gestational weight gain.^62–64^ This mechanism is often discussed in the context of maternal anthropometric risk measures being more strongly associated with child obesity than paternal measures, which we did not observe here.^9^^,^^14–16^^,^^25^ However, this does not rule out some contribution of intrauterine pathways to observed associations. Genetic variants have also been implicated in the development of obesity, although their total contribution is understood to be low at the population level.^65^^,^^66^ Detailed, longitudinal studies are required to more clearly elucidate the relative contribution of these pathways to the development of child obesity in this population.

This study was based on a large subset of individuals participating in a population-based health survey in Malaysia. By contrast to other studies,^8^^,^^10^^,^^11^^,^^20^^,^^23^^,^^26^ all our analyses were based on objective measures of anthropometric and cardiometabolic risk factors rather than on self-report. Missing data were minimal with respect to child characteristics, and ranged around 5–10% for parental characteristics, with no consistent differences between individuals with missing versus non-missing data. This study was based on individuals living in a defined geographical region in peninsular Malaysia, and the prevalence of overweight and obesity among adults and children reported here is greater than previously reported national estimates, which might suggest some limits to generalizability.^33^ As with all cross-sectional studies, our analyses limit inferences about causality. Furthermore, they provide estimates of association specifically in terms of current child and parental anthropometric and cardiometabolic measures, which may not fully reflect associations using historical measures.^16^^,^^18^ Furthermore, despite our large sample size, we may not have had the required statistical resolution to detect interactive effects, which may be distinct in this population. Nonetheless, this study adds to the currently scarce evidence from Asia on intergenerational influences on child obesity.

In all, we show that parental obesity is strongly and independently associated with increased risk of child obesity in this Malaysian population. Our findings indicate the value of understanding intergenerational contributions to the risk of child obesity. They provide a basis for further prospective studies based in Asian populations to establish the temporality and the pathways underlying the associations observed here. Evidence from such studies is essential to inform strategies that can more effectively address the growing burden of child obesity in this region.

## Supplementary Data

Supplementary data are available at *IJE* online.

## Funding

SEACO is funded by: the office of the Vice Provost Research, Monash University Australia; the office of the Deputy Dean Research, Faculty of Medicine, Nursing and Health Sciences, Monash University Australia; the Monash University Malaysia Campus; and the Jeffrey Cheah School of Medicine and Health Sciences. SEACO is an associate member of the INDEPTH Network. This work was supported by the Wellcome Trust (grant number 098051). M.S. is supported by the National Institute for Health Research Cambridge Biomedical Research Centre (UK). U.P. is supported by the Dr Herchel Smith Fellowship.

**Key Messages**

- This study adds to the limited evidence from Asia on the relation between parental anthropometric and cardiometabolic risk factors and child obesity.
- Child obesity was independently associated with parental obesity in this population. There was no consistent evidence of associations between parental cardiometabolic risk factors and child obesity.
- Associations observed were not modified by parental or child sex.
- These findings have implications regarding the design of interventions to address child obesity in this region.

## Supplementary Material

Supplementary Data
