# Supplementary material for: Anthropometric and cardiometabolic risk factors in parents and child obesity in Segamat, Malaysia
Source: Int J Epidemiol. 2017 Jun 29;46(5):1523–32. doi: 10.1093/ije/dyx114 (PMC5837730; doi:10.1093/ije/dyx114)
Supplement: Supplementary Data [file dyx114_ije-2016-09-1053-file004.docx]

**Anthropometric and cardiometabolic risk factors in parents and child obesity in Segamat, Malaysia**

**Supplementary Tables**

| **Table S1. Comparison of maternal and child characteristics by missing status for blood pressure or random blood glucose data.** | | | | | | | | | | | | | | | | | | |  |
| --- | --- | --- | --- | --- | --- | --- | --- | --- | --- | --- | --- | --- | --- | --- | --- | --- | --- | --- | --- |
|  |  | **Systolic blood pressure** | | | | |  | **Diastolic blood pressure** | | | | |  | **Random blood glucose data** | | | | |  |
|  |  | **Missing** | | **Non-missing** | | ***P*** |  | **Missing** | | **Non-missing** | | ***P*** |  | **Missing** | | **Non-missing** | | ***P*** |  |
|  |  |  |  |  |  |  |  |  |  |  |  |  |  |  |  |  |  |  | |
| **N, %** |  | 284 | (11.1) | 2286 | (88.9) |  |  | 281 | (10.9) | 2289 | (89.1) |  |  | 267 | (10.4) | 2303 | (89.6) |  | |
|  |  |  |  |  |  |  |  |  |  |  |  |  |  |  |  |  |  |  | |
| **Eldest child's BMI z-score** |  | 0.4 | (1.4) | 0.3 | (1.5) | 0.236 |  | 0.4 | (1.4) | 0.3 | (1.5) | 0.273 |  | 0.4 | (1.4) | 0.3 | (1.5) | 0.221 | |
|  |  |  |  |  |  |  |  |  |  |  |  |  |  |  |  |  |  |  | |
| **Age, years, mean (SD)** |  | 32.6 | (6.1) | 49.1 | (9.2) | <0.001 |  | 32.4 | (5.8) | 49.1 | (9.2) | <0.001 |  | 31.5 | (3.2) | 49.1 | (9.2) | <0.001 | |
|  |  |  |  |  |  |  |  |  |  |  |  |  |  |  |  |  |  |  | |
| **Body mass index, kg/m^2^, mean (SD)** |  | 26.3 | (5.1) | 27.5 | (5.4) | 0.001 |  | 26.5 | (5.3) | 27.5 | (5.3) | 0.006 |  | 26.3 | (5.2) | 27.5 | (5.3) | <0.001 | |
| **Waist circumference, cm, mean (SD)** |  | 83.2 | (9.7) | 87.0 | (11.0) | <0.001 |  | 83.2 | (10.0) | 87.0 | (11.0) | <0.001 |  | 82.9 | (9.6) | 87.0 | (11.0) | <0.001 | |
| **Systolic blood pressure, mmHg, mean (SD)** |  | n/a | | | | |  | 142.5 | (21.8) | 129.0 | (18.3) | 0.011 |  | 127.7 | (12.6) | 129.1 | (18.4) | 0.892 | |
| **Diastolic blood pressure, mmHg, mean (SD)** |  | 90.4 | (20.2) | 78.7 | (10.6) | <0.001 |  | n/a | | | | |  | 86.2 | (5.4) | 78.8 | (10.7) | 0.231 | |
| **Random blood glucose, mmol/L, mean (SD)** |  | 10.3 | (5.5) | 7.9 | (3.6) | 0.003 |  | 9.6 | (5.3) | 7.9 | (3.6) | 0.050 |  | n/a | | | | | |
|  |  |  |  |  |  |  |  |  |  |  |  |  |  |  |  |  |  |  | |
| **Education level attained, n (%)** |  |  |  |  |  |  |  |  |  |  |  |  |  |  |  |  |  |  | |
| None |  | 5 | (7.5) | 62 | (92.5) |  |  | 3 | (4.5) | 64 | (95.5) |  |  | 3 | (4.5) | 64 | (95.5) |  | |
| Attended/completed Primary |  | 44 | (5.8) | 714 | (94.2) |  |  | 42 | (5.5) | 716 | (94.5) |  |  | 37 | (4.9) | 721 | (95.1) |  | |
| Attended/completed Secondary |  | 212 | (13.8) | 1330 | (86.3) |  |  | 212 | (13.8) | 1330 | (86.3) |  |  | 203 | (13.2) | 1339 | (86.8) |  | |
| Attended/completed Tertiary/Diploma |  | 19 | (20.9) | 72 | (79.1) | <0.001 |  | 19 | (20.9) | 72 | (79.1) | <0.001 |  | 19 | (20.9) | 72 | (79.1) | <0.001 | |
| Missing |  | 4 | (3.6) | 108 | (96.4) |  |  | 5 | (4.5) | 107 | (95.5) |  |  | 5 | (4.5) | 107 | (95.5) |  | |
|  |  |  |  |  |  |  |  |  |  |  |  |  |  |  |  |  |  |  | |
| **Employment in past 30 days, n (%)** |  |  |  |  |  |  |  |  |  |  |  |  |  |  |  |  |  |  | |
| Student/Not working |  | 9 | (5.6) | 152 | (94.4) |  |  | 10 | (6.2) | 151 | (93.8) |  |  | 9 | (5.6) | 152 | (94.4) |  | |
| Casual/Part-time |  | 12 | (9.4) | 116 | (90.6) |  |  | 13 | (10.2) | 115 | (89.8) |  |  | 12 | (9.4) | 116 | (90.6) |  | |
| Full time |  | 70 | (15.4) | 385 | (84.6) |  |  | 70 | (15.4) | 385 | (84.6) |  |  | 68 | (15.0) | 387 | (85.1) |  | |
| Self-employed/Housewife |  | 193 | (10.7) | 1608 | (89.3) |  |  | 188 | (10.4) | 1613 | (89.6) |  |  | 178 | (9.9) | 1623 | (90.1) |  | |
| Pensions |  | 0 | (0.0) | 22 | (100.0) | 0.002 |  | 0 | (0.0) | 22 | (100.0) | 0.003 |  | 0 | (0.0) | 22 | (100.0) | 0.002 | |
| Missing |  | 0 | (0.0) | 3 | (100.0) |  |  | 0 | (0.0) | 3 | (100.0) |  |  | 0 | (0.0) | 3 | (100.0) |  | |
|  |  |  |  |  |  |  |  |  |  |  |  |  |  |  |  |  |  |  | |
| *P* values are reported for differences in variable means (continuous variables), or differences in proportions across variable categories (categorical variables), between non-missing and missing. | | | | | | | | | | | | | | | | | | |  |
| Student's t-test was used to compare continuous variables, and Pearson's chi-squared and Fisher's exact tests were used to compare categorical variables (Fisher's exact for cell frequencies less than five). | | | | | | | | | | | | | | | | | | |  |

| **Table S2. Household and subdistrict level clustering of cardiometabolic risk factors in the study population.** | | | | | | | | |
| --- | --- | --- | --- | --- | --- | --- | --- | --- |
|  |  | **Unadjusted intra-class correlation coefficient (95% confidence interval)** | | | | | | |
|  |  | **Household level** | | |  | **Subdistrict level** | | |
|  |  |  |  |  |  |  |  |  |
| **Children** |  |  |  |  |  |  |  |  |
| **Body mass index-for-age** |  | 0.30 | (0.27, | 0.34) |  | 0.00 | (0.00, | 0.00) |
|  |  |  |  |  |  |  |  |  |
| **Adults** |  |  |  |  |  |  |  |  |
| **Body mass index** |  | 0.23 | (0.19, | 0.26) |  | 0.02 | (0.00, | 0.05) |
| **Waist circumference** |  | 0.10 | (0.08, | 0.13) |  | 0.01 | (0.00, | 0.02) |
| **Systolic blood pressure** |  | 0.12 | (0.07, | 0.16) |  | 0.02 | (0.00, | 0.06) |
| **Diastolic blood pressure** |  | 0.18 | (0.13, | 0.22) |  | 0.03 | (0.00, | 0.08) |
| **Random blood glucose** |  | 0.11 | (0.06, | 0.15) |  | 0.01 | (0.00, | 0.03) |
|  |  |  |  |  |  |  |  |  |

| **Table S3. Correlation between maternal and paternal anthropometric and cardiometabolic risk measures.** | | |
| --- | --- | --- |
|  | **Pearson's correlation coefficient** | ***P*** |
| **Body mass index** | 0.180 | <0.001 |
| **Waist circumference** | 0.165 | <0.001 |
| **Systolic blood pressure** | 0.185 | <0.001 |
| **Diastolic blood pressure** | 0.219 | <0.001 |
| **Random blood glucose** | 0.094 | <0.001 |

| **Table S4. Proportion of children obese by child characteristic.** | | | | | | |
| --- | --- | --- | --- | --- | --- | --- |
|  |  | **Non-obese** | | **Obese** | | ***P*** |
|  |  |  |  |  |  |  |
| **N** |  | 4117 | (85.7) | 689 | (14.3) |  |
|  |  |  |  |  |  |  |
| **Sex, n (%)** |  |  |  |  |  |  |
| Male |  | 1962 | (83.1) | 400 | (16.9) |  |
| Female |  | 2155 | (88.2) | 289 | (17.8) | <0.001 |
|  |  |  |  |  |  |  |
| **Age, years, mean (SD)** |  | 12.9 | (3.8) | 11.7 | (3.7) | <0.001 |
|  |  |  |  |  |  |  |
| **Ethnicity, n (%)** |  |  |  |  |  |  |
| Malay |  | 2771 | (85.1) | 485 | (14.9) |  |
| Indian |  | 405 | (86.9) | 61 | (13.9) |  |
| Chinese |  | 829 | (87.5) | 119 | (12.6) |  |
| Bumiputera/Orang Asli |  | 70 | (81.4) | 16 | (18.6) |  |
| Other |  | 26 | (81.3) | 6 | (18.8) | 0.227 |
| Missing |  | 16 | (88.9) | 2 | (11.1) |  |
|  |  |  |  |  |  |  |
| **Birth order, n (%)** |  |  |  |  |  |  |
| 1 |  | 1750 | (85.4) | 299 | (14.6) |  |
| 2 |  | 1267 | (86.4) | 200 | (13.6) |  |
| 3 |  | 672 | (84.8) | 120 | (15.2) |  |
| 4 |  | 279 | (86.1) | 45 | (13.9) |  |
| 5 |  | 104 | (85.9) | 17 | (14.1) |  |
| 6 |  | 30 | (83.3) | 6 | (16.7) |  |
| 7+ |  | 15 | (88.2) | 2 | (11.8) | 0.963 |
|  | | | | | | |
| *P* values are reported for differences in variable means (continuous variables), or differences in proportions across variable categories (categorical variables), between non-obese and obese. | | | | | | |
| Student's t-test was used to compare continuous variables, and Pearson's chi-squared and Fisher's exact tests were used to compare categorical variables (Fisher’s exact for categories with cell frequencies of less than five). | | | | | | |

| **Table S5. Risk ratios for child obesity associated with number of parents with a specific cardiometabolic risk factor.** | | | | | | | | | | | | |
| --- | --- | --- | --- | --- | --- | --- | --- | --- | --- | --- | --- | --- |
|  |  |  |  | **One parent^1^** | | | |  | **Both parents** | | | |
| **Parental risk factor** |  | **N** |  | **Risk ratio (95% confidence interval)** | | | ***P*** |  | **Risk ratio (95% confidence interval)** | | | ***P*** |
|  |  |  |  |  |  |  |  |  |  |  |  |  |
| **Overweight** |  |  |  |  |  |  |  |  |  |  |  |  |
| Model 1 |  | 2136 |  | 1.16 | (0.83, | 1.63) | 0.382 |  | 1.43 | (0.99, | 2.08) | 0.057 |
| Model 2 |  | 2126 |  | 1.18 | (0.84, | 1.67) | 0.342 |  | 1.44 | (0.99, | 2.09) | 0.054 |
| Model 3 |  | 2126 |  | 1.21 | (0.86, | 1.70) | 0.275 |  | 1.48 | (1.02, | 2.14) | 0.038 |
| Model 4 |  | 2003 |  | 1.12 | (0.80, | 1.58) | 0.513 |  | 1.54 | (1.06, | 2.24) | 0.025 |
| **Obese** |  |  |  |  |  |  |  |  |  |  |  |  |
| Model 1 |  | 3347 |  | 1.44 | (1.17, | 1.77) | <0.001 |  | 2.34 | (1.81, | 3.04) | <0.001 |
| Model 2 |  | 3335 |  | 1.42 | (1.16, | 1.75) | 0.001 |  | 2.32 | (1.78, | 3.01) | <0.001 |
| Model 3 |  | 3335 |  | 1.45 | (1.18, | 1.78) | <0.001 |  | 2.30 | (1.77, | 2.98) | <0.001 |
| Model 4 |  | 3149 |  | 1.44 | (1.15, | 1.80) | 0.001 |  | 2.38 | (1.82, | 3.10) | <0.001 |
| **Centrally obese** |  |  |  |  |  |  |  |  |  |  |  |  |
| Model 1 |  | 3215 |  | 1.52 | (1.09, | 2.12) | 0.013 |  | 2.03 | (1.46, | 2.82) | <0.001 |
| Model 2 |  | 3203 |  | 1.51 | (1.08, | 2.10) | 0.015 |  | 2.08 | (1.50, | 2.89) | <0.001 |
| Model 3 |  | 3203 |  | 1.58 | (1.13, | 2.22) | 0.008 |  | 2.16 | (1.55, | 3.02) | <0.001 |
| Model 4 |  | 3032 |  | 1.62 | (1.14, | 2.29) | 0.007 |  | 2.18 | (1.55, | 3.07) | <0.001 |
| **Systolic hypertension^2^** |  |  |  |  |  |  |  |  |  |  |  |  |
| Model 1 |  | 3093 |  | 1.32 | (1.06, | 1.63) | 0.012 |  | 1.68 | (1.25, | 2.26) | 0.001 |
| Model 2 |  | 3087 |  | 1.32 | (1.06, | 1.64) | 0.012 |  | 1.65 | (1.23, | 2.22) | 0.001 |
| Model 3 |  | 2937 |  | 1.25 | (1.00, | 1.57) | 0.049 |  | 1.50 | (1.08, | 2.09) | 0.015 |
| Model 4 |  | 2762 |  | 1.22 | (0.96, | 1.55) | 0.101 |  | 1.45 | (1.02, | 2.07) | 0.039 |
| **Diastolic hypertension^2^** |  |  |  |  |  |  |  |  |  |  |  |  |
| Model 1 |  | 3112 |  | 1.25 | (1.00, | 1.55) | 0.047 |  | 1.43 | (0.98, | 2.10) | 0.064 |
| Model 2 |  | 3106 |  | 1.22 | (0.98, | 1.52) | 0.071 |  | 1.41 | (0.96, | 2.07) | 0.080 |
| Model 3 |  | 2957 |  | 1.13 | (0.90, | 1.41) | 0.307 |  | 1.21 | (0.84, | 1.74) | 0.299 |
| Model 4 |  | 2782 |  | 1.10 | (0.87, | 1.39) | 0.434 |  | 1.20 | (0.83, | 1.72) | 0.339 |
| **Hyperglycemic^3^** |  |  |  |  |  |  |  |  |  |  |  |  |
| Model 1 |  | 3145 |  | 1.37 | (1.06, | 1.76) | 0.015 |  | 2.39 | (1.45, | 3.94) | 0.001 |
| Model 2 |  | 3139 |  | 1.37 | (1.06, | 1.76) | 0.015 |  | 2.36 | (1.43, | 3.91) | 0.001 |
| Model 3 |  | 2937 |  | 1.24 | (0.94, | 1.63) | 0.133 |  | 2.12 | (1.23, | 3.65) | 0.007 |
| Model 4 |  | 2762 |  | 1.36 | (1.02, | 1.81) | 0.035 |  | 2.28 | (1.27, | 4.12) | 0.006 |
|  |  |  |  |  |  |  |  |  |  |  |  |  |
| Model 1: Unadjusted. | | | | | | | | | | | | |
| Model 2: Adjusted for child's birth order and ethnicity. | | | | | | | | | | | | |
| Model 3: Adjusted as Model 2 and for maternal age and paternal age. | | | | | | | | | | | | |
| Model 4: Adjusted as Model 3 and for maternal and paternal education and employment. | | | | | | | | | | | | |
| ^1^One parent refers to either the mother or the father having the specific risk factor. | | | | | | | | | | | | |
| ^2^Models exploring systolic and diastolic blood pressure also included adjustments for maternal BMI and random blood glucose (Model 3 onwards). | | | | | | | | | | | | |
| ^3^Models exploring random blood glucose also included adjustments for maternal and paternal BMI and systolic blood pressure (Model 3 onwards). | | | | | | | | | | | | |

| **Table S6. Risk ratios for child obesity associated with number of parents having a specific cardiometabolic risk factor, overall and stratified by child sex.** | | | | | | | | | | | | |
| --- | --- | --- | --- | --- | --- | --- | --- | --- | --- | --- | --- | --- |
|  |  |  |  | **One parent^1^** | | | |  | **Both parents** | | | |
| **Population or subgroup; parental risk factor** |  | **N** |  | **Risk ratio (95% confidence interval)** | | | ***P*** |  | **Risk ratio (95% confidence interval)** | | | ***P*** |
|  |  |  |  |  |  |  |  |  |  |  |  |  |
| **Overall** |  |  |  |  |  |  |  |  |  |  |  |  |
| **Overweight** |  | 2003 |  | 1.12 | (0.80, | 1.58) | 0.513 |  | 1.54 | (1.06, | 2.24) | 0.025 |
| **Obese** |  | 3149 |  | 1.44 | (1.15, | 1.80) | 0.001 |  | 2.38 | (1.82, | 3.10) | <0.001 |
| **Centrally obese** |  | 3032 |  | 1.62 | (1.14, | 2.29) | 0.007 |  | 2.18 | (1.55, | 3.07) | <0.001 |
| **Systolic hypertension^1^** |  | 2762 |  | 1.22 | (0.96, | 1.55) | 0.101 |  | 1.45 | (1.02, | 2.07) | 0.039 |
| **Diastolic hypertension^1^** |  | 2782 |  | 1.10 | (0.87, | 1.39) | 0.434 |  | 1.20 | (0.83, | 1.72) | 0.339 |
| **Hyperglycemic^2^** |  | 2762 |  | 1.36 | (1.02, | 1.81) | 0.035 |  | 2.28 | (1.27, | 4.12) | 0.006 |
|  |  |  |  |  |  |  |  |  |  |  |  |  |
| **Daughters** |  |  |  |  |  |  |  |  |  |  |  |  |
| **Overweight** |  | 1031 |  | 1.38 | (0.80, | 2.37) | 0.247 |  | 2.00 | (1.09, | 3.66) | 0.026 |
| **Obese** |  | 1640 |  | 1.58 | (1.14, | 2.20) | 0.006 |  | 2.76 | (1.88, | 4.06) | <0.001 |
| **Centrally obese** |  | 1590 |  | 1.58 | (0.94, | 2.66) | 0.086 |  | 2.74 | (1.64, | 4.59) | <0.001 |
| **Systolic hypertension^2^** |  | 1447 |  | 1.20 | (0.84, | 1.71) | 0.313 |  | 1.46 | (0.83, | 2.55) | 0.189 |
| **Diastolic hypertension^2^** |  | 1451 |  | 1.33 | (0.94, | 1.89) | 0.108 |  | 1.60 | (0.93, | 2.75) | 0.089 |
| **Hyperglycemic^3^** |  | 1447 |  | 1.54 | (1.03, | 2.30) | 0.037 |  | 1.35 | (0.58, | 3.14) | 0.491 |
|  |  |  |  |  |  |  |  |  |  |  |  |  |
| **Sons** |  |  |  |  |  |  |  |  |  |  |  |  |
| **Overweight** |  | 1016 |  | 1.26 | (0.84, | 1.88) | 0.256 |  | 1.38 | (0.90, | 2.12) | 0.139 |
| **Obese** |  | 1559 |  | 1.36 | (1.05, | 1.76) | 0.021 |  | 2.10 | (1.43, | 3.09) | <0.001 |
| **Centrally obese** |  | 1489 |  | 1.76 | (1.18, | 2.63) | 0.006 |  | 1.98 | (1.31, | 2.99) | 0.001 |
| **Systolic hypertension^2^** |  | 1360 |  | 1.39 | (1.03, | 1.87) | 0.033 |  | 1.46 | (0.95, | 2.25) | 0.082 |
| **Diastolic hypertension^2^** |  | 1376 |  | 1.00 | (0.73, | 1.36) | 0.980 |  | 0.86 | (0.46, | 1.62) | 0.644 |
| **Hyperglycemic^3^** |  | 1360 |  | 1.16 | (0.81, | 1.67) | 0.417 |  | 2.62 | (1.26, | 5.43) | 0.010 |
|  |  |  |  |  |  |  |  |  |  |  |  |  |
| Models stratified by child sex were adjusted for child's ethnicity, birth order, and maternal and paternal age and employment, and maternal education. | | | | | | | | | | | | |
| ^1^One parent refers to either the mother or the father having the specific risk factor. | | | | | | | | | | | | |
| ^2^Models exploring systolic and diastolic blood pressure also included adjustments for maternal and paternal BMI and random blood glucose. | | | | | | | | | | | | |
| ^3^Models exploring random blood glucose also included adjustments for maternal and paternal BMI and systolic blood pressure. | | | | | | | | | | | | |

| **Table S7. Per-unit increase in risk for child obesity associated with number of parents having a specific cardiometabolic risk factor.** | | | | | | | |
| --- | --- | --- | --- | --- | --- | --- | --- |
| **Population or subgroup; parental risk factor** |  | **N** |  | **Per unit increase (95% confidence interval)** | | | ***P*** |
|  |  |  |  |  |  |  |  |
| **Overall** |  |  |  |  |  |  |  |
| **Overweight** |  | 2003 |  | 1.26 | (1.04, | 1.53) | 0.016 |
| **Obese** |  | 3149 |  | 1.53 | (1.34, | 1.74) | <0.001 |
| **Centrally obese** |  | 3032 |  | 1.43 | (1.23, | 1.65) | <0.001 |
| **Systolic hypertension^1^** |  | 2762 |  | 1.24 | (1.05, | 1.46) | 0.010 |
| **Diastolic hypertension^1^** |  | 2782 |  | 1.13 | (0.96, | 1.32) | 0.132 |
| **Hyperglycemic^2^** |  | 2762 |  | 1.42 | (1.12, | 1.79) | 0.004 |
|  |  |  |  |  |  |  |  |
| **Daughters** |  |  |  |  |  |  |  |
| **Overweight** |  | 1031 |  | 1.42 | (1.05, | 1.92) | 0.024 |
| **Obese** |  | 1640 |  | 1.65 | (1.36, | 1.99) | <0.001 |
| **Centrally obese** |  | 1590 |  | 1.67 | (1.33, | 2.09) | <0.001 |
| **Systolic hypertension^1^** |  | 1447 |  | 1.20 | (0.94, | 1.54) | 0.139 |
| **Diastolic hypertension^1^** |  | 1451 |  | 1.29 | (1.02, | 1.62) | 0.032 |
| **Hyperglycemic^2^** |  | 1447 |  | 1.36 | (1.00, | 1.85) | 0.051 |
|  |  |  |  |  |  |  |  |
| **Sons** |  |  |  |  |  |  |  |
| **Overweight** |  | 1016 |  | 1.17 | (0.95, | 1.45) | 0.130 |
| **Obese** |  | 1159 |  | 1.42 | (1.19, | 1.70) | <0.001 |
| **Centrally obese** |  | 1489 |  | 1.33 | (1.12, | 1.58) | 0.001 |
| **Systolic hypertension^1^** |  | 1360 |  | 1.26 | (1.04, | 1.53) | 0.020 |
| **Diastolic hypertension^1^** |  | 1376 |  | 0.96 | (0.76, | 1.21) | 0.722 |
| **Hyperglycemic^2^** |  | 1360 |  | 1.32 | (0.98, | 1.79) | 0.066 |
|  |  |  |  |  |  |  |  |
| Likelihood ratio tests suggested no departure from linearity when comparing models when the primary exposure (number of parents with the risk factor) as a continuous versus a categorical variable. | | | | | | | |
| Models using the full study population were adjusted for child's ethnicity, birth order, and maternal and paternal age, education and employment. To facilitate convergence, the fully-adjusted model for parental overweight was adjusted for maternal education only. | | | | | | | |
| Models stratified by child sex were adjusted for child's ethnicity, birth order, and maternal and paternal age and employment, and maternal education. | | | | | | | |
| ^1^Models exploring systolic and diastolic blood pressure also included adjustments for maternal and paternal BMI and random blood glucose. | | | | | | | |
| ^2^Models exploring random blood glucose also included adjustments for maternal and paternal BMI and systolic blood pressure. | | | | | | | |

| **Table S8. Risk ratios for child obesity associated with maternal and paternal cardiometabolic risk factors.** | | | | | | | | | | | | |
| --- | --- | --- | --- | --- | --- | --- | --- | --- | --- | --- | --- | --- |
|  |  | **Maternal** | | | | |  | **Paternal** | | | | |
| **Parental risk factor** |  | **N** | **Risk ratio (95% confidence interval)** | | | ***P*** |  | **N** | **Risk ratio (95% confidence interval)** | | | ***P*** |
|  |  |  |  |  |  |  |  |  |  |  |  |  |
| **Overweight** |  |  |  |  |  |  |  |  |  |  |  |  |
| Model 1 |  | 3405 | 1.48 | (1.20, | 1.82) | <0.001 |  | 2837 | 1.30 | (1.04, | 1.61) | 0.019 |
| Model 2 |  | 3388 | 1.49 | (1.21, | 1.83) | <0.001 |  | 2827 | 1.31 | (1.06, | 1.63) | 0.014 |
| Model 3 |  | 3243 | 1.47 | (1.19, | 1.81) | <0.001 |  | 2742 | 1.34 | (1.07, | 1.68) | 0.010 |
| Model 4 |  | 2232 | 1.31 | (1.01, | 1.70) | 0.043 |  | 2383 | 1.30 | (1.02, | 1.66) | 0.033 |
| Model 5 |  | 2184 | 1.31 | (0.99, | 1.71) | 0.055 |  | 2301 | 1.25 | (0.97, | 1.61) | 0.080 |
| **Obese** |  |  |  |  |  |  |  |  |  |  |  |  |
| Model 1 |  | 4704 | 1.90 | (1.63, | 2.21) | <0.001 |  | 3414 | 1.60 | (1.30, | 1.96) | <0.001 |
| Model 2 |  | 4686 | 1.89 | (1.62, | 2.21) | <0.001 |  | 3402 | 1.56 | (1.27, | 1.91) | <0.001 |
| Model 3 |  | 4486 | 1.93 | (1.65, | 2.26) | <0.001 |  | 3311 | 1.56 | (1.27, | 1.92) | <0.001 |
| Model 4 |  | 3054 | 1.61 | (1.32, | 1.98) | <0.001 |  | 2900 | 1.36 | (1.10, | 1.69) | 0.005 |
| Model 5 |  | 3001 | 1.64 | (1.33, | 2.02) | <0.001 |  | 2805 | 1.34 | (1.07, | 1.67) | 0.011 |
| **Centrally obese** |  |  |  |  |  |  |  |  |  |  |  |  |
| Model 1 |  | 4617 | 1.95 | (1.56, | 2.42) | <0.001 |  | 3336 | 1.28 | (1.06, | 1.54) | 0.010 |
| Model 2 |  | 4599 | 2.03 | (1.62, | 2.55) | <0.001 |  | 3324 | 1.30 | (1.07, | 1.57) | 0.007 |
| Model 3 |  | 4413 | 2.07 | (1.65, | 2.62) | <0.001 |  | 3234 | 1.28 | (1.05, | 1.55) | 0.013 |
| Model 4 |  | 2997 | 1.89 | (1.42, | 2.51) | <0.001 |  | 2835 | 1.20 | (0.98, | 1.48) | 0.080 |
| Model 5 |  | 2946 | 1.87 | (1.41, | 2.49) | <0.001 |  | 2740 | 1.17 | (0.94, | 1.45) | 0.161 |
| **Systolic hypertension^1^** |  |  |  |  |  |  |  |  |  |  |  |  |
| Model 1 |  | 4345 | 1.14 | (0.95, | 1.37) | 0.151 |  | 3355 | 1.34 | (1.09, | 1.64) | 0.005 |
| Model 2 |  | 4232 | 1.00 | (0.82, | 1.21) | 0.963 |  | 3248 | 1.37 | (1.10, | 1.70) | 0.004 |
| Model 3 |  | 4038 | 1.01 | (0.83, | 1.23) | 0.938 |  | 3161 | 1.32 | (1.06, | 1.66) | 0.014 |
| Model 4 |  | 2810 | 1.14 | (0.89, | 1.46) | 0.308 |  | 2857 | 1.30 | (1.03, | 1.64) | 0.029 |
| Model 5 |  | 2762 | 1.12 | (0.87, | 1.45) | 0.372 |  | 2762 | 1.31 | (1.02, | 1.67) | 0.032 |
| **Diastolic hypertension^1^** |  |  |  |  |  |  |  |  |  |  |  |  |
| Model 1 |  | 4357 | 1.18 | (0.97, | 1.44) | 0.106 |  | 3359 | 1.23 | (0.98, | 1.55) | 0.080 |
| Model 2 |  | 4245 | 0.99 | (0.81, | 1.21) | 0.935 |  | 3251 | 1.17 | (0.93, | 1.48) | 0.188 |
| Model 3 |  | 4053 | 1.05 | (0.86, | 1.29) | 0.619 |  | 3162 | 1.10 | (0.87, | 1.40) | 0.429 |
| Model 4 |  | 2828 | 1.08 | (0.84, | 1.37) | 0.555 |  | 2857 | 1.13 | (0.89, | 1.45) | 0.321 |
| Model 5 |  | 2780 | 1.08 | (0.84, | 1.38) | 0.545 |  | 2764 | 1.12 | (0.87, | 1.44) | 0.379 |
| **Hyperglycemic^2^** |  |  |  |  |  |  |  |  |  |  |  |  |
| Model 1 |  | 4382 | 1.57 | (1.25, | 1.97) | <0.001 |  | 3374 | 1.34 | (0.99, | 1.83) | 0.061 |
| Model 2 |  | 4232 | 1.33 | (1.06, | 1.68) | 0.015 |  | 3248 | 1.29 | (0.91, | 1.81) | 0.150 |
| Model 3 |  | 4038 | 1.44 | (1.14, | 1.82) | 0.002 |  | 3161 | 1.31 | (0.93, | 1.84) | 0.120 |
| Model 4 |  | 2810 | 1.46 | (1.08, | 1.98) | 0.014 |  | 2857 | 1.29 | (0.91, | 1.82) | 0.159 |
| Model 5 |  | 2762 | 1.52 | (1.12, | 2.07) | 0.008 |  | 2762 | 1.32 | (0.93, | 1.88) | 0.125 |
|  |  |  |  |  |  |  |  |  |  |  |  |  |
| Model 1: unadjusted. | | | | | | | | | | | | |
| Model 2: adjusted for child's ethnicity and birth order, and parent's age. | | | | | | | | | | | | |
| Model 3: adjusted as Model 2 and for parent's education and employment. | | | | | | | | | | | | |
| Model 4: adjusted as Model 3 and for other parent's age, body mass index, systolic blood pressure and random blood glucose. | | | | | | | | | | | | |
| Model 5: adjusted as Model 4 and for other parent's education and employment. | | | | | | | | | | | | |
| ^1^Models exploring hypertension were additionally adjusted for parent's body mass index and random blood glucose. | | | | | | | | | | | | |
| ^2^Models exploring hyperglycemia were additionally adjusted for parent's body mass index and systolic blood pressure. | | | | | | | | | | | | |

| **Table S9. Risk ratios for child obesity associated with maternal and paternal cardiometabolic risk factors, overall and stratified by child sex.** | | | | | | | | | | | | |
| --- | --- | --- | --- | --- | --- | --- | --- | --- | --- | --- | --- | --- |
|  |  | **Maternal** | | | | |  | **Paternal** | | | | |
| **Population or subgroup; parental risk factor** |  | **N** | **Risk ratio (95% confidence interval)** | | | ***P*** |  | **N** | **Risk ratio (95% confidence interval)** | | | ***P*** |
|  |  |  |  |  |  |  |  |  |  |  |  |  |
| **Overall** |  |  |  |  |  |  |  |  |  |  |  |  |
| **Overweight** |  | 2184 | 1.31 | (0.99, | 1.71) | 0.055 |  | 2301 | 1.25 | (0.97, | 1.61) | 0.080 |
| **Obese** |  | 3001 | 1.64 | (1.33, | 2.02) | <0.001 |  | 2805 | 1.34 | (1.07, | 1.67) | 0.011 |
| **Centrally obese** |  | 2946 | 1.87 | (1.41, | 2.49) | <0.001 |  | 2740 | 1.17 | (0.94, | 1.45) | 0.161 |
| **Systolic hypertension^1^** |  | 2762 | 1.12 | (0.87, | 1.45) | 0.372 |  | 2762 | 1.31 | (1.02, | 1.67) | 0.032 |
| **Diastolic hypertension^1^** |  | 2780 | 1.08 | (0.84, | 1.38) | 0.545 |  | 2764 | 1.12 | (0.87, | 1.44) | 0.379 |
| **Hyperglycemic^2^** |  | 2762 | 1.52 | (1.12, | 2.07) | 0.008 |  | 2762 | 1.32 | (0.93, | 1.88) | 0.125 |
|  |  |  |  |  |  |  |  |  |  |  |  |  |
| **Daughters** |  |  |  |  |  |  |  |  |  |  |  |  |
| **Overweight** |  | 1135 | 2.07 | (1.32, | 3.25) | 0.001 |  | 1186 | 1.06 | (0.72, | 1.54) | 0.775 |
| **Obese** |  | 1563 | 1.87 | (1.38, | 2.55) | <0.001 |  | 1472 | 1.28 | (0.92, | 1.79) | 0.148 |
| **Centrally obese** |  | 1539 | 2.90 | (1.77, | 4.75) | <0.001 |  | 1444 | 1.14 | (0.82, | 1.58) | 0.439 |
| **Systolic hypertension^1^** |  | 1447 | 1.06 | (0.71, | 1.56) | 0.787 |  | 1447 | 1.36 | (0.95, | 1.97) | 0.097 |
| **Diastolic hypertension^1^** |  | 1445 | 1.31 | (0.92, | 1.87) | 0.141 |  | 1443 | 1.33 | (0.93, | 1.91) | 0.122 |
| **Hyperglycemic^2^** |  | 1447 | 1.42 | (0.92, | 2.19) | 0.117 |  | 1447 | 1.30 | (0.76, | 2.21) | 0.339 |
|  |  |  |  |  |  |  |  |  |  |  |  |  |
| **Sons** |  |  |  |  |  |  |  |  |  |  |  |  |
| **Overweight** |  | 1096 | 1.03 | (0.76, | 1.41) | 0.837 |  | 1157 | 1.34 | (0.99, | 1.82) | 0.056 |
| **Obese** |  | 1487 | 1.40 | (1.08, | 1.80) | 0.011 |  | 1378 | 1.54 | (1.15, | 2.06) | 0.004 |
| **Centrally obese** |  | 1454 | 1.50 | (1.09, | 2.07) | 0.014 |  | 1341 | 1.23 | (0.95, | 1.60) | 0.123 |
| **Systolic hypertension^1^** |  | 1360 | 1.17 | (0.85, | 1.63) | 0.331 |  | 1360 | 1.35 | (1.01, | 1.82) | 0.043 |
| **Diastolic hypertension^1^** |  | 1370 | 0.84 | (0.60, | 1.18) | 0.323 |  | 1366 | 1.05 | (0.74, | 1.50) | 0.779 |
| **Hyperglycemic^2^** |  | 1360 | 1.46 | (0.99, | 2.17) | 0.058 |  | 1360 | 1.19 | (0.77, | 1.83) | 0.433 |
|  |  |  |  |  |  |  |  |  |  |  |  |  |
| Models using the full study population were adjusted for child's ethnicity, birth order, parent's age, education and employment, and for other parent's age, body mass index, systolic blood pressure, random blood glucose, education and employment. | | | | | | | | | | | | |
| Models stratified by child sex were adjusted for child's ethnicity, birth order, parent's age, education (mother only) and employment, and for other parent's age, body mass index, systolic blood pressure, random blood glucose, education (mother only) and employment. | | | | | | | | | | | | |
| ^1^Models exploring blood pressure were additionally adjusted for parent's body mass index and random blood glucose. | | | | | | | | | | | | |
| ^2^Models exploring random blood glucose were additionally adjusted for parent's body mass index and systolic blood pressure. | | | | | | | | | | | | |

| **Table S10. Exploration of interactions between parental or child sex and parental risk factors in Poisson regression models.** | | | | | | |
| --- | --- | --- | --- | --- | --- | --- |
| **Parental risk factor** |  |  |  | ***P* for likelihood ratio test comparing fully adjusted models with versus without interaction terms** | | |
|  |  | **N** |  | **Parent's sex^1^** | **Child's sex^2^** | **Parent's sex and child's sex^3^** |
|  |  |  |  |  |  |  |
| **Overweight** |  | 4485 |  | 0.988 | 0.639 | 0.868 |
| **Obese** |  | 5806 |  | 0.913 | 0.592 | 0.797 |
| **Centrally obese** |  | 5686 |  | 0.279 | 0.382 | 0.224 |
| **Systolic hypertension^4^** |  | 5524 |  | 1.000 | 0.935 | 0.993 |
| **Diastolic hypertension^4^** |  | 5544 |  | 1.000 | 0.180 | 0.399 |
| **Hyperglycemic^5^** |  | 5524 |  | 0.872 | 0.752 | 0.814 |
|  |  |  |  |  |  |  |
| ^1^Fully adjusted models were adjusted for parent's sex, child's ethnicity, birth order, parent's age, education and employment, and for other parent's age, body mass index, systolic blood pressure, random blood glucose, education and employment. Additional interaction term: parent's sex x parent's risk factor. | | | | | | |
| ^2^Fully adjusted models were adjusted as in (1) and additionally for child's sex. Additional interaction term: child's sex x parent's risk factor. | | | | | | |
| ^3^Fully adjusted models were adjusted as in (2). Additional interaction terms: parent's sex x parent's risk factor and child's sex x parent's risk factor. | | | | | | |
| ^4^Models exploring blood pressure were additionally adjusted for parent's body mass index and random blood glucose. | | | | | | |
| ^5^Models exploring random blood glucose were additionally adjusted for parent's body mass index and systolic blood pressure. | | | | | | |
| The dataset was reshaped to allow investigation of interactions. Hence, additionally to the household level, all models were also adjusted for clustering at the child's level. | | | | | | |

| **Table S11. Regression coefficients for association of maternal and paternal cardiometabolic risk factors with child BMI z-score.** | | | | | | | | | | | | |
| --- | --- | --- | --- | --- | --- | --- | --- | --- | --- | --- | --- | --- |
|  |  | **Maternal** | | | | |  | **Paternal** | | | | |
| **Parental risk factor** |  | **N** | **β (95% confidence interval)** | | | ***P*** |  | **N** | **β (95% confidence interval)** | | | ***P*** |
|  |  |  |  |  |  |  |  |  |  |  |  |  |
| **Body mass index, kg/m^2^** |  |  |  |  |  |  |  |  |  |  |  |  |
| Model 1 |  | 4704 | 0.053 | (0.044, | 0.062) | <0.001 |  | 3414 | 0.043 | (0.030, | 0.056) | <0.001 |
| Model 2 |  | 4486 | 0.058 | (0.048, | 0.067) | <0.001 |  | 3311 | 0.044 | (0.030, | 0.057) | <0.001 |
| Model 3 |  | 3001 | 0.046 | (0.034, | 0.059) | <0.001 |  | 2805 | 0.032 | (0.017, | 0.046) | <0.001 |
| **Waist circumference, cm** |  |  |  |  |  |  |  |  |  |  |  |  |
| Model 1 |  | 4617 | 0.022 | (0.018, | 0.027) | <0.001 |  | 3336 | 0.016 | (0.011, | 0.022) | <0.001 |
| Model 2 |  | 4413 | 0.026 | (0.022, | 0.031) | <0.001 |  | 3234 | 0.018 | (0.012, | 0.024) | <0.001 |
| Model 3 |  | 2946 | 0.023 | (0.017, | 0.028) | <0.001 |  | 2740 | 0.013 | (0.007, | 0.019) | <0.001 |
| **Systolic blood pressure^1^, mmHg** |  |  |  |  |  |  |  |  |  |  |  |  |
| Model 1 |  | 4345 | 0.005 | (0.002, | 0.008) | 0.001 |  | 3355 | 0.006 | (0.003, | 0.009) | <0.001 |
| Model 2 |  | 4038 | 0.003 | (0.000, | 0.006) | 0.082 |  | 3161 | 0.004 | (0.001, | 0.008) | 0.016 |
| Model 3 |  | 2762 | 0.003 | (-0.001, | 0.007) | 0.127 |  | 2762 | 0.004 | (0.000, | 0.007) | 0.055 |
| **Diastolic blood pressure^1^, mmHg** |  |  |  |  |  |  |  |  |  |  |  |  |
| Model 1 |  | 4357 | 0.008 | (0.004, | 0.013) | 0.001 |  | 3359 | 0.006 | (0.001, | 0.011) | 0.026 |
| Model 2 |  | 4053 | 0.002 | (-0.003, | 0.007) | 0.385 |  | 3162 | 0.002 | (-0.003, | 0.007) | 0.480 |
| Model 3 |  | 2780 | 0.004 | (-0.002, | 0.009) | 0.196 |  | 2764 | 0.001 | (-0.004, | 0.007) | 0.612 |
| **Random blood glucose^2^, mmol/L** |  |  |  |  |  |  |  |  |  |  |  |  |
| Model 1 |  | 4382 | 0.032 | (0.017, | 0.047) | <0.001 |  | 3374 | 0.022 | (0.003, | 0.040) | 0.022 |
| Model 2 |  | 4038 | 0.028 | (0.012, | 0.044) | 0.001 |  | 3161 | 0.015 | (-0.004, | 0.035) | 0.126 |
| Model 3 |  | 2762 | 0.037 | (0.018, | 0.056) | <0.001 |  | 2762 | 0.013 | (-0.008, | 0.033) | 0.218 |
|  |  |  |  |  |  |  |  |  |  |  |  |  |
| β values represent the change in child BMI z-score per unit increase in parental risk factor measure. | | | | | | | | | | | | |
| Model 1: unadjusted. | | | | | | | | | | | | |
| Model 2: adjusted for child's ethnicity and birth order, and for parent's age, education and employment. | | | | | | | | | | | | |
| Model 3: adjusted as Model 2 and for other parent's age, body mass index, systolic blood pressure, random blood glucose, education and employment. | | | | | | | | | | | | |
| ^1^Models exploring blood pressure were additionally adjusted for parent's body mass index and random blood glucose. | | | | | | | | | | | | |
| ^2^Models exploring random blood glucose were additionally adjusted for parent's body mass index and systolic blood pressure. | | | | | | | | | | | | |

| **Table S12. Regression coefficients for association of maternal and paternal cardiometabolic risk factors with child BMI z-score, overall and stratified by child sex.** | | | | | | | | | | | | |
| --- | --- | --- | --- | --- | --- | --- | --- | --- | --- | --- | --- | --- |
|  |  | **Maternal** | | | | |  | **Paternal** | | | | |
| **Population or subgroup; parental risk factor** |  | **N** | **β (95% confidence interval)** | | | ***P*** |  | **N** | **β (95% confidence interval)** | | | ***P*** |
|  |  |  |  |  |  |  |  |  |  |  |  |  |
| **Overall** |  |  |  |  |  |  |  |  |  |  |  |  |
| **Body mass index, kg/m^2^** |  | 3001 | 0.046 | (0.034, | 0.059) | <0.001 |  | 2805 | 0.032 | (0.017, | 0.046) | <0.001 |
| **Waist circumference, cm** |  | 2946 | 0.023 | (0.017, | 0.028) | <0.001 |  | 2740 | 0.013 | (0.007, | 0.019) | <0.001 |
| **Systolic blood pressure^1^, mmHg** |  | 2762 | 0.003 | (-0.001, | 0.007) | 0.127 |  | 2762 | 0.004 | (0.000, | 0.007) | 0.055 |
| **Diastolic blood pressure^1^, mmHg** |  | 2780 | 0.004 | (-0.002, | 0.009) | 0.196 |  | 2764 | 0.001 | (-0.004, | 0.007) | 0.612 |
| **Random blood glucose^2^, mmol/L** |  | 2762 | 0.037 | (0.018, | 0.056) | <0.001 |  | 2762 | 0.013 | (-0.008, | 0.033) | 0.218 |
|  |  |  |  |  |  |  |  |  |  |  |  |  |
| **Daughters** |  |  |  |  |  |  |  |  |  |  |  |  |
| **Body mass index, kg/m^2^** |  | 1541 | 0.048 | (0.033, | 0.063) | <0.001 |  | 1452 | 0.025 | (0.009, | 0.041) | 0.002 |
| **Waist circumference, cm** |  | 1517 | 0.023 | (0.016, | 0.030) | <0.001 |  | 1424 | 0.011 | (0.004, | 0.018) | 0.001 |
| **Systolic blood pressure^1^, mmHg** |  | 1427 | 0.002 | (-0.003, | 0.006) | 0.512 |  | 1427 | 0.004 | (0.000, | 0.008) | 0.068 |
| **Diastolic blood pressure^1^, mmHg** |  | 1435 | 0.002 | (-0.005, | 0.008) | 0.605 |  | 1423 | 0.003 | (-0.004, | 0.009) | 0.376 |
| **Random blood glucose^2^, mmol/L** |  | 1427 | 0.043 | (0.022, | 0.065) | 0.000 |  | 1427 | 0.024 | (-0.001, | 0.049) | 0.061 |
|  |  |  |  |  |  |  |  |  |  |  |  |  |
| **Sons** |  |  |  |  |  |  |  |  |  |  |  |  |
| **Body mass index, kg/m^2^** |  | 1460 | 0.039 | (0.022, | 0.057) | <0.001 |  | 1353 | 0.041 | (0.018, | 0.064) | <0.001 |
| **Waist circumference, cm** |  | 1429 | 0.019 | (0.011, | 0.028) | <0.001 |  | 1316 | 0.017 | (0.007, | 0.027) | 0.001 |
| **Systolic blood pressure^1^, mmHg** |  | 1335 | 0.004 | (-0.002, | 0.009) | 0.179 |  | 1335 | 0.004 | (-0.002, | 0.009) | 0.216 |
| **Diastolic blood pressure^1^, mmHg** |  | 1345 | 0.004 | (-0.004, | 0.013) | 0.336 |  | 1341 | 0.001 | (-0.007, | 0.009) | 0.853 |
| **Random blood glucose^2^, mmol/L** |  | 1335 | 0.028 | (-0.003, | 0.058) | 0.077 |  | 1335 | 0.005 | (-0.022, | 0.033) | 0.696 |
|  |  |  |  |  |  |  |  |  |  |  |  |  |
| β values represent the change in child BMI z-score per unit increase in parental risk factor measure. | | | | | | | | | | | | |
| Models using the full study population were adjusted for child's ethnicity, birth order, parent's age, education and employment, and for other parent's age, body mass index, systolic blood pressure, random blood glucose, education and employment. | | | | | | | | | | | | |
| Models stratified by child sex were adjusted as above. | | | | | | | | | | | | |
| ^1^Models exploring blood pressure were additionally adjusted for parent's body mass index and random blood glucose. | | | | | | | | | | | | |
| ^2^Models exploring random blood glucose were additionally adjusted for parent's body mass index and systolic blood pressure. | | | | | | | | | | | | |
